# Neural correlates underlying impaired memory facilitation and suppression of negative material in depression

**DOI:** 10.1038/srep37556

**Published:** 2016-11-18

**Authors:** Dandan Zhang, Hui Xie, Yunzhe Liu, Yuejia Luo

**Affiliations:** 1 Institute of Affective and Social Neuroscience, Shenzhen University, Shenzhen 518060, China

## Abstract

Previous behavioral studies demonstrated that depressed individuals have difficulties in forgetting unwanted, especially negative, event. However, inconsistent results still exit and the neural mechanism of this phenomenon has not been investigated. This study examined the intentional memory facilitation/suppression of negative and neutral materials in depression using Think/No-Think paradigm. We found that compared with nondepressed group, depressed group recalled more negative items, irrespective of either "Think" or "No-Think" instructions. Accordingly, the frontal N2 (reflecting voluntary memory inhibition) and parietal late positive component (LPC) (reflecting conscious recollection) showed deflection for negative items in depressed compared with nondepressed participants. On the one hand, the reduced N2 for negative "No-Think" items indicated that depressed individuals have low motivation to suppress negative items so intentional forgetting is less successful for mood-congruent events. On the other hand, the enhanced LPC for negative "Think" items suggested that negative memories are excessively revisited by depressed participants (compared with nondepressed ones) due to their mood-congruent and intrusive nature. Thus we demonstrated that depressed individuals show behavioral and ERP deviations from healthy controls for both voluntary suppression and conscious retrieval of negative memory; the two abnormalities of memory control together contribute to the difficulties in forgetting negative material in depression.

Recurrent and uncontrollable negative cognitions play a critical role in the etiology and maintenance of depression^1^. Compared with nondepressed individuals, depressed individuals excessively attend to and remember negatively valenced stimuli/events^2^,^3^. Recent literatures suggest that the excessively enhanced memory for negative material in depression is not only caused by the "bottom-up" deficit of biased selective attention to negativity, but also due to the "top-down" impairment of executive control that disables patients from removing irrelevant negative material from memory^4^,^5^. Numerous studies have demonstrated that, compared with nondepressed counterparts, depressed individuals have more difficulties in excluding the unwanted memory from awareness^6^,^7^,^8^,^9^,^10^,^11^,^12^. Such memory suppression deficits may induce sustained flashbacks associated with a gloomy thinking trend, and the enhanced gloomy feeling, in turn, makes negative memories more easily to be retrieved, resulting in a vicious circle of mood-congruent recall and prolonged depressive mood^10^,^13^,^14^. In this context, therefore, it is proposed that the inability to suppress negative memory may contribute largely to maladaptive ruminative tendency—a hallmark of depressive disorder^4^,^15^. Investigating the voluntary memory suppression and its associated neural mechanism in depressed population can, on the one hand, provides novel insight into the symptoms and the pathology of the disorder, and on the other hand, helps us to reconsider the link between cognitive control and emotion, thus optimizing the clinical treatment for patients.

One frequently employed paradigm to examine the intentional memory suppression is the Think/No-Think (T/NT) task^16^,^17^. In this paradigm, participants learn cue-target pairs before they enter the T/NT phase, where only the cues are presented and participants are instructed to suppress retrieval of the related target to some cues (No-Think condition) while elaborate retrieval of the related target to other cues (Think condition). Studies found that stopping retrieval in the presence of cues increases the probability of later forgetting of related targets, and engages brain regions associated with active control (lateral prefrontal cortex, LPFC, and anterior cingulated cortex, ACC); meanwhile, it reduces the neural activity in the medial-temporal declarative memory system (hippocampus)^17^,^18^,^19^. Furthermore, the increased LPFC activity correlates with the decreased hippocampus activity, and this correlation predicts the behavioral success of memory suppression^19^,^20^. When using emotional materials as cues or targets in the T/NT task, researchers observed in non-psychotic populations that "suppression-induced forgetting" is significant for memories associated with emotionally negative but not positive material^21^, and that, relative to memory for neutral items, memory for negative items is enhanced in both Think^22^ and No-Think conditions^23^.

In the past years, the detrimental effect of depression on controlling the intrusion of unwanted thoughts has been investigated in several behavioral T/NT studies. In particular, Hertel and Gerstel^8^ observed a close correlation between the severity of rumination and inhibitory difficulties; compared with nondepressed individuals, depressed individuals recalled higher percentages of targets in the No-Think condition (see also in Hertel & Mahan^24^). In another research, Dieler *et al*.^7^ investigated the intentional memory suppression in 71 volunteers, finding that high brooding tendency predicted worse suppression performance and that this effect was more obvious for negative, compared with neutral, memories. Additional evidence for reduced inhibition for negative items in depression was provided by Joormann *et al*.^10^, who found that individuals with major depressive disorder, in contrast with control participants, had more difficulties in forgetting negative items by directly suppressing them from retrieval.

Although these behavioral studies demonstrated a deficit of suppression-induced forgetting in depression, inconsistent results still exist. For example, the success of intentional memory suppression in the T/NT is usually reflected by a lower recall rate of suppressed targets compared with the recall rate of baseline targets (i.e., targets which have been neither suppressed nor retrieved)^16^. However, while many studies failed to produce below-baseline suppression for negative and/or neutral materials in depressed individuals^7^,^8^,^10^,^24^, Joormann *et al*.^25^ found that depressed patients successfully forgot negative targets and showed enhanced effect of below-baseline forgetting as more opportunities for suppression had been provided. Thus the first rationale for the present study emerged from the disparate findings in depressed individuals. Furthermore, one of the advantages of the T/NT paradigm is to examine both voluntary remembering (Think) and forgetting (No-Think) in a single study. However, previous T/NT studies in depression either did not compare the "Think" performance between groups (high *vs*. low depression, or patients *vs*. controls)^7^,^10^,^25^ or did not find any difference in Think condition between groups^8^,^24^. More importantly, the neural mechanism underlying the impaired performance in the T/NT task has not been investigated. Thus the aim of the current study was to use event-related potential (ERP) technique to examine the neural correlates of the impaired memory facilitation/suppression in depressed individuals so as to help advance theories of depression and improve treatments for this disorder.

On the basis of previous studies in healthy subjects, two ERP components have been consistently found to be related to memory facilitatory/inhibitory processing. The early T/NT effect lies in the frontal N2 component with a maximum between 200 and 300 ms after cue presentation; the N2 is enhanced for No-Think trials *vs*. Think trials and is proposed to be associated with an inhibitory process that attempts to avoid memory retrieval^17^,^23^,^26^,^27^,^28^,^29^,^30^. Since the T/NT paradigm is a modification of the Go/Nogo paradigm, this early negativity is thought by many researchers to have a shared neural basis with the Nogo-N2 component^26^,^29^, the latter of which reflects an inhibitory control process that suppresses unwanted motor responses^31^. Another ERP component that be sensitive to the T/NT task is the parietal late positive component (LPC), with a maximum between 500 and 800 ms after cue onset; Think items usually evoke a larger LPC than No-Think items^23^,^26^,^27^,^29^,^32^,^33^. In general, the LPC is proposed to reflect an episodic memory (EM) effect and indexes conscious recollection^34^,^35^,^36^. It has been known that enhanced LPC predicts retrieval success^27^,^37^,^38^, and that this ERP marker can be downregulated during attempts to stop recollection^29^,^33^. Besides, previous memory studies using emotional stimuli have discovered that negative memories elicit larger LPC amplitudes than neutral memories, suggesting an emotion-induced enhancement of memory recognition^23^,^39^,^40^.

In the current study, we speculated that the cognitive control mechanism underlies both retrieval facilitation and retrieval stopping may be disrupted in depression, leading to difficulties in forgetting mood-congruent memories. Based on the aforementioned behavioral studies in depression, we expected that the less successful attempt to avoid negative memory retrieval may reduce the amplitudes of the frontal N2 in No-Think items. Meanwhile, the difficulties in forgetting mood-congruent contents is also likely caused by excessive memory retrieval of negative stimuli/events; and this procedure may produce enhanced LPC amplitudes in negatively valenced Think items.

## Methods

### Participants

In order to exclude the potential influence of psychiatric medications on results^41^,^42^, this study examined the dysfunction of memory suppression in individuals with depression tendency rather than depressed patients. Furthermore, in view of the fact that anxiety and depressive symptoms are highly comorbid^43^,^44^,^45^, and that some researches indicate a correlation between anxiety and inability of memory suppression^7^, we only recruited participants with high-trait anxiety in this study, i.e., the memory suppression deficit was investigated in comparison between the depressed individuals with anxiety and the nondepressed individuals with anxiety.

All the freshman students (n = 6725) in Shenzhen University were required to complete the Chinese versions of the Beck Depression Inventory Second Edition (BDI-II^46^) and the Trait form of Spielberger's State-Trait Anxiety Inventory (STAI-T^47^,^48^) (first-level screen). In this sample, individuals with STAI-T scores in the upper 25% of the distribution were considered as high-trait anxiety subjects^49^,^50^ and passed into a second-level screen. Among the high-trait anxiety sample, individuals with BDI-II scores ≤13 were labeled as nondepressed subjects, whereas individuals scored >13 in BDI-II were labeled as depressed subjects. From those who met these criteria, we randomly recruited 50 students as paid participants (25 depressed and 25 nondepressed ones). There was no significant difference between the two groups with respect to age, handedness and STAI-T scores (Table 1).

Exclusion criteria for both groups were (1) any Axis I and II disorders according to the Diagnostic and Statistical Manual (DSM-IV^51^); (2) severe depression (BDI-II ≥ 29); (3) seizure disorder; (4) history of head injury with possible neurological sequelae, and (5) substance abuse or dependence in the past six months.

Written informed consent was obtained from every participant prior to the experiment. The experimental protocol was approved by the Ethics Committee of Shenzhen University and this study was performed strictly in accordance with the approved guidelines.

### Stimuli

The T/NT task was performed using faces as cues and pictures as targets^19^,^22^,^23^, since compared with the frequently used word-word combinations, pictorial stimuli can lead to more salient memory representations^52^ and have more clinical relevance in psychiatric symptoms^53^,^54^.

Forty-eight neutral faces were used as cues, with equal number of facial pictures between males and females. These faces were previously rated as having a neutral expression by 20 participants in a pilot study using a 9-point scale (valence = 5.02 ± 0.16, arousal = 5.17 ± 0.18).

Forty-eight pictures (24 negative and 24 neutral ones) were selected from the International Affective Picture System (IAPS^55^) as targets. All the pictures in this study were also used in the study of Depue *et al*.^22^. To eliminate grouping effects, Depue *et al*.^22^ ensured that these pictures had as minimal relatedness in content as possible. Each picture had been assessed for its valence and arousal on a 9-point scale with a large sample of Chinese participants in a previous survey. Consistent with Depue *et al*.^22^, the negative and neutral pictures were at a median level of arousal (negative = 5.19 ± 0.11, neutral = 5.12 ± 0.11, t(46) = 0.44, *p* = 0.661), but the two categories differed significantly in valence (negative = 2.57 ± 0.16, neutral = 5.00 ± 0.15, t(46) = −11.0, *p* < 0.001).

### Procedure

All the participants were required to perform the BDI-II and the STAI-T again before they entered the experimental room.

**Figure 1:**
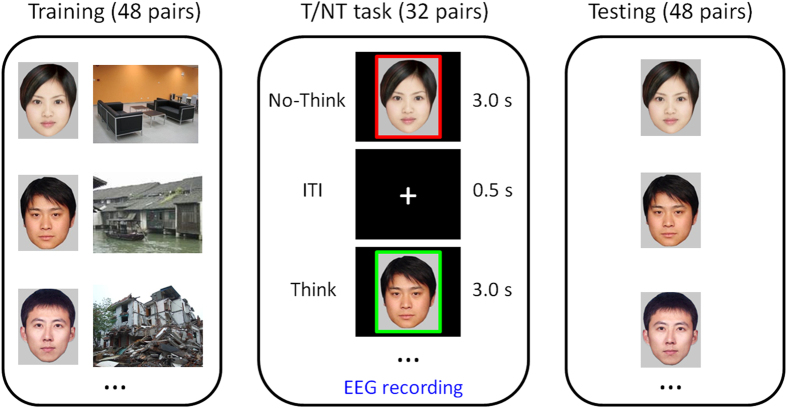
Illustration of the Think/No-Think (T/NT) task. In this study, 48 pictures (24 negative and 24 neutral ones) were selected from the International Affective Picture System (IAPS^55^) as the targets of the 48 cue-target pairs. Here we used another three pictures taken by ourselves to replace the target pictures used in the study for the reason of copyright.

The T/NT paradigm was composed of three phases: training, T/NT task, and testing (Fig. 1)^16^,^17^,^19^,^22^,^32^,^33^.

In the training phase, participants were required to remember 48 face-picture pairs. Participants first viewed each of 48 pairs (4 s per pair) and were then shown only the faces and asked to produce a description of 3–5 words for the pictures associated with the faces. The description was then judged as correct or incorrect by one experimenter. This procedure continued until participants could remember the face-picture pairs with an accuracy of >96% over all 48 pairs. The training phase lasted for about 25 to 40 min in each participant.

In the T/NT phase, participants saw the faces from 32 of the 48 pairs, with 8 pairs in each of the four conditions (think of negative target, no-think of negative target, think of neutral target, and no-think of neutral target). Each trial began with a fixation cross for 500 ms, followed by a face for 3 s (3.0° × 3.6° visual angle). The color of the border around a face indicated the task: green for think trials and red for no-think trials, or red for think trials and green for no-think trials. The assignment of colors to think/no-think tasks was counterbalanced across participants. The instruction for the think condition was "Recall the picture previously associated with the face," whereas the instruction for the no-think condition was "Prevent the previously associated picture from entering consciousness (keep yourself from think about the picture by keeping your mind completely blank)." To obtain an ideal suppression effect, participants were instructed to pay full attention to the cue and not to think of anything else. The 32 faces were presented in a pseudo-random sequence, and each face appeared 10 times during the T/NT phase. The 16 faces not shown in this phase served as a behavioral baseline.

In order to ensure participants understood and followed the instructions, they were required to complete a brief questionnaire, at the beginning and the middle of the T/NT phase. Three questions in the questionnaire were: (1) when you saw the No-Think cue, were you able to avoid thinking about the target picture associated with it? (2) when you saw the Think cue, were you able to think about the target picture associated with it? and (3) did you actively push the No-Think target out of mind if it did come to mind after seeing the No-Think cue? The experimenter would communicate with participants if any abnormity was found in the questionnaire, so noncompliance with instructions for suppression can be ruled out.

In the memory test phase, the 48 faces were presented successively with a pseudo-random order. Participants were required to produce a description of 3–5 words for the pictures previously associated with the faces. These descriptions were recorded and then scored as correct or incorrect by two independent experimenters (inter-rater reliability was above 0.9 for each participant).

The interval between the T/NT phase and the memory test phase was an hour. Participants were required to perform an irrelevant-task (delay discounting) during this interval (the task was about 40 min).

### EEG recording and analysis

During the T/NT phase, brain electrical activity was recorded referentially against left mastoid and off-line re-referenced to the average of the left and right mastoids, by a 64-channel amplifier with a sampling frequency of 250 Hz (Brain Products, Gilching, Germany). Electroencephalography (EEG) data were collected with electrode impedances kept below 5 kΩ. Ocular artifacts were removed from EEGs using a regression procedure implemented in NeuroScan software (Scan 4.3).

The recorded EEG data were filtered (0.01–30 Hz) and segmented beginning 200 ms prior to the onset of stimulus and lasting for 1700 ms. Then epochs were baseline-corrected with respect to the mean voltage over the 200 ms preceding the onset of stimulus, followed by averaging in association with experimental conditions, irrespective of recall accuracy^19^. Trials contaminated with large artifacts (peak-to-peak deflection exceeded ± 100 μV) were excluded from the averaging. As a result, 147 ± 10 trials and 145 ± 11 trials were left in each subject for the think and no-think condition, respectively (there were totally 160 trials in each condition). Trial numbers did not show significant difference between conditions and between groups.

This study focused on the ERPs elicited by Think negative, Think neutral, No-Think negative, and No-think neutral cues in the two subject groups. We analyzed the average amplitudes of the frontal N2 and parietal LPC across different sets of electrodes according to grand-mean ERP topographies and relevant literatures^23^,^26^,^32^,^34^. The N2 amplitude was calculated as the average amplitude at the electrode sites of Fz, FCz, F1 and F2 between 190 to 230 ms after the onset of cues. The LPC amplitude was calculated as the average amplitude at the electrode sites of Pz, CPz, P1 and P2 between 500 to 800 ms post stimulus.

### Statistics

Descriptive data were presented as mean ± standard error. The significance level was set at 0.05.

Repeated-measures ANOVA was performed on behavioral and ERP measurements, with instruction (Think vs. No-Think in the ERP data, whereas Think, No-Think, and baseline in the recall rate) and the emotion category of pictures (negative vs. neutral) as within-subject factors, and group (depressed vs. nondepressed) as the between-subject factor. Greenhouse-Geisser correction for ANOVA tests was used whenever appropriate. *Post-hoc* testing of significant main effects was conducted using Bonferroni method. Significant interactions were analyzed using simple effects model.

## Results

### Recall rate in the memory test

**Figure 2:**
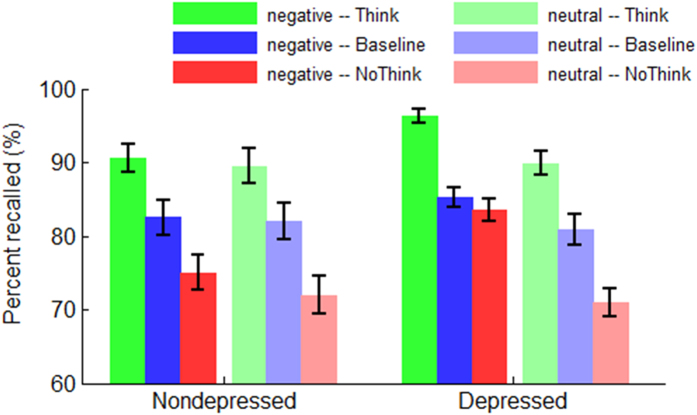
The recall rate in the post-testing. Bars represent ± standard error of the mean.

The interaction of emotion by group was significant (*F*(1,48) = 13.5, *p* = 0.001, 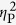 = 0.219; Fig. 2). Depressed individuals recalled more negative targets than nondepressed ones (*F*(1,48) = 5.64, *p* = 0.022; depressed = 88.0 ± 1.50%, nondepressed = 83.0 ± 1.50%) while the recall rate of neutral targets did not significantly differ between the two groups (*F*(1,48) < 1; depressed = 80.6 ± 1.93%, nondepressed = 81.2 ± 1.93%). Furthermore, it was found that depressed individuals recalled more negative than neutral targets (*F*(1,24) = 47.2, *p* < 0.001); however the recall rate between negative and neutral targets did not achieve significant level in nondepressed individuals (*F*(1,24) = 2.82, *p* = 0.100).

The interaction of instruction by emotion was significant (*F*(2,96) = 4.26, *p* = 0.018, 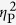 = 0.081). Compared to the Think condition (*F*(1,48) = 7.37, *p* = 0.009; negative = 93.4 ± 1.07%, neutral = 89.7 ± 1.41%) and the baseline condition (*F*(1,48) = 2.99, *p* = 0.090; negative = 83.8 ± 1.41%, neutral = 81.4 ± 1.58%), participants recalled much more negative than neutral items with the No-Think instruction (*F*(1,48) = 40.0, *p* < 0.001; negative = 79.2 ± 1.42%, neutral = 71.5 ± 1.59%). In addition, the recall rate showed a bigger difference between Think, baseline and No-Think conditions when participants recalled neutral targets (*F*(2,96) = 114, *p* < 0.001), while showed a smaller difference when participants recalled negative targets (*F*(2,96) = 57.8, *p* < 0.001).

The main effect of instruction was significant (*F*(2,96) = 181, *p* < 0.001, 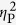 = 0.790). Think targets were recalled with a higher rate (91.6 ± 1.05%, *p*s < 0.001) than both baseline targets (82.6 ± 1.32%) and No-Think targets (75.4 ± 1.38%). Meanwhile, No-Think targets were recalled with a lower rate than baseline targets (*p* < 0.001).

The main effect of emotion was significant (*F*(1,48) = 36.6, *p* < 0.001, 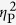 = 0.432). Negative targets were recalled with a higher rate (85.5 ± 1.06%) compared with neutral targets (80.9 ± 1.36%).

Although the interaction of instruction by emotion by group was not significant (*F*(2,96) = 1.97, *p* = 0.147, 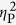 = 0.039), the interaction of instruction by group achieved significant level when only considering the negative condition (*F*(2,96) = 4.26, *p* = 0.019, 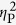 = 0.081). Within this two-way interaction, the depressed individuals recalled more Think (*F*(1,48) = 7.34, *p* = 0.009; depressed = 96.3 ± 1.51%, nondepressed = 90.5 ± 1.51%) and No-Think negative targets (*F*(1,48) = 8.89, *p* = 0.004; depressed = 83.5 ± 2.02%, nondepressed = 75.0 ± 2.02%) than nondepressed individuals while the recall rate of negative targets did not significantly differ between the two groups in the baseline condition (*F*(1,48) < 1; depressed = 84.2 ± 2.00%, nondepressed = 83.5 ± 2.00%). Also, the two-way interaction (instruction × group) for the negative targets indicated that the task instruction influenced the two groups in different patterns (nondepressed: *F*(2,48) = 33.4, depressed: *F*(2,48) = 28.6, *p*s < 0.001). In particular, while the nondepressed individuals recalled more baseline targets than No-Think targets (*t*(24) = 3.65, *p* = 0.001), the depressed individuals recalled similar numbers of negative targets in baseline and No-Think conditions (*t*(24) = 0.420, *p* = 0.678).

### ERPs

#### N2

**Figure 3:**
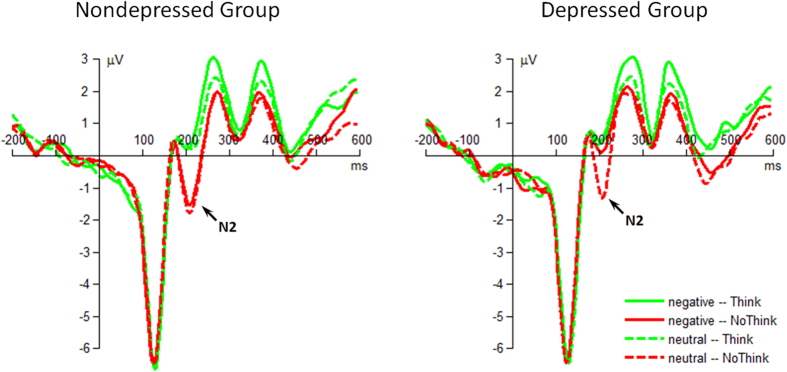
The grand-mean ERP waveforms of the frontal N2 component. Waveforms were calculated by averaging the data at the electrodes of Fz, FCz, F1 and F2.

The interaction of instruction by emotion by group was significant (*F*(1,48) = 4.16, *p* = 0.047, 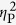 = 0.080; Fig. 3). For the group effect, depressed individuals had smaller N2 (0.31 ± 0.33 μV) than nondepressed participants (−1.48 ± 0.33 μV) when the cues were associated with negative targets in No-Think condition (*F*(1,48) = 14.7, *p* < 0.001). However, this group difference did not achieve significant level in both Think (*F*(1,48) < 1; nondepressed = 0.47 ± 0.30 μV, depressed = 0.63 ± 0.30 μV) and No-Think conditions (*F*(1,48) = 1.06, *p* = 0.308; nondepressed = −1.62 ± 0.33 μV, depressed = −1.15 ± 0.33 μV) for neutral targets, nor in Think condition for negative targets (*F*(1,48) < 1; nondepressed = 0.43 ± 0.32 μV, depressed = 0.69 ± 0.32 μV). For the emotion effect, the N2 showed smaller amplitudes when depressed individuals suppressed the negative, compared to neutral, targets (*F*(1,48) = 21.3, *p* < 0.001); however, this N2 difference caused by emotion did not achieve significant level when depressed individuals were instructed to Think the targets (*F*(1,48) < 1), nor when nondepressed participants were instructed to Think (*F*(1,48) < 1) or No-Think the targets (*F*(1,48) < 1). For the instruction effect, both groups had larger N2 amplitudes in response to No-Think than Think instruction when the cues were associated with neutral targets (nondepressed: *F*(1,48) = 69.7, *p* < 0.001; depressed *F*(1,48) = 50.7, *p* < 0.001). However this N2 difference caused by instruction only achieved significant level in nondepressed individuals when the cues were associated with negative targets (nondepressed: *F*(1,48) = 49.0, *p* < 0.001; depressed: *F*(1,48) = 1.98, *p* = 0.166).

In addition, the interaction of emotion by group was significant (*F*(1,48) = 6.05, *p* = 0.018, 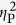 = 0.112). And the interaction of instruction by emotion was significant (*F*(1,48) = 6.96, *p* = 0.011, 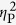 = 0.127).

The main effect of instruction was significant (*F*(1,48) = 199, *p* < 0.001, 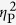 = 0.806). No-Think instruction evoked larger N2 (−0.99 ± 0.20 μV) compared with Think instruction (0.56 ± 0.20 μV).

The main effect of emotion was significant (*F*(1,48) = 7.83, *p* = 0.007, 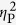 = 0.140). Negative targets evoked smaller N2 (−0.01 ± 0.21 μV) than neutral targets (−0.42 ± 0.20 μV) did.

#### LPC

**Figure 4:**
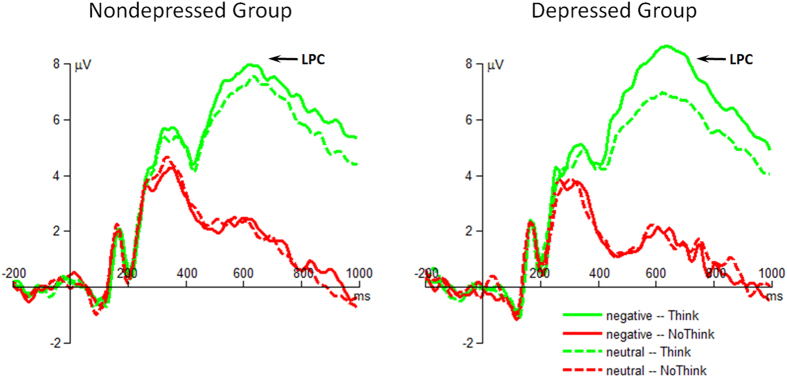
The grand-mean ERP waveforms of the parietal LPC component. Waveforms were calculated by averaging the data at the electrodes of Pz, CPz, P1 and P2.

The interaction of instruction by emotion by group was significant (*F*(1,48) = 5.28, *p* = 0.026, 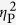 = 0.099; Fig. 4). For the group effect, depressed individuals had larger LPC (8.33 ± 0.51 μV) than nondepressed participants (6.82 ± 0.51 μV) when the cues were associated with negative targets in Think condition (*F*(1,48) = 4.36, *p* = 0.042). However, this group difference did not achieve significant level in both Think (*F*(1,48) < 1; nondepressed = 6.39 ± 0.50 μV, depressed = 6.12 ± 0.50 μV) and No-Think conditions (*F*(1,48) < 1; nondepressed = 2.16 ± 0.38 μV, depressed = 1.82 ± 0.38 μV) for neutral targets, nor in No-Think condition for negative targets (*F*(1,48) < 1; nondepressed = 2.22 ± 0.44 μV, depressed = 1.80 ± 0.44 μV). For the emotion effect, the LPC showed larger amplitudes when depressed individuals were instructed to Think the negative, compared to neutral, targets (*F*(1,48) = 17.6, *p* < 0.001); however, this LPC difference caused by emotion did not achieve significant level when depressed individuals were instructed to No-Think the targets (*F*(1,48) < 1), nor when nondepressed participants were instructed to Think (*F*(1,48) < 1) or No-Think the targets (*F*(1,48) < 1). For the instruction effect, although Think instruction always evoked larger LPC amplitudes than No-Think instruction, this LPC difference caused by instruction was much bigger when depressed individuals were presented with the cues associated with negative targets (negative for depressed: *F*(1,48) = 168, *p* < 0.001; negative for nondepressed: *F*(1,48) = 83.3, *p* < 0.001; neutral for depressed: *F*(1,48) = 65.7, *p* < 0.001; neutral for nondepressed: *F*(1,48) = 63.5, *p* < 0.001).

In addition, the interaction of instruction by emotion was significant (*F*(1,48) = 10.3, *p* = 0.002, 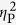 = 0.177).

The main effect of instruction was significant (*F*(1,48) = 260, *p* < 0.001, 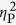 = 0.844). Think instruction evoked much larger LPC (6.91 ± 0.30 μV) compared with No-Think instruction (2.00 ± 0.25 μV).

The main effect of emotion was significant (*F*(1,48) = 6.18, *p* = 0.016, 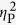 = 0.144). Negative targets evoked larger LPC (4.79 ± 0.29 μV) than neutral targets (4.12 ± 0.25 μV) did.

## Discussion

Depression is characterized by frequent occurrence of negative thoughts and memories^1^,^56^, which plays a causal role in maintaining the disorder^10^. To answer the question why depressed individuals have difficulties in forgetting negative material, this study examined the intentional memory facilitation/suppression of negative and neutral materials in depressed participants using T/NT paradigm. For neural items, depressed and nondepressed individuals showed comparable behavioral and ERP results, indicating that the brains of the two groups work in a similar manner when neutral memory is consciously retrieved or suppressed. Likewise, for negative items and in the baseline condition, memory recall test revealed no significant difference between the two groups (see also in Hertel and Gerstle^8^), suggesting there are no deficits in depressed individuals when negative stimuli/events are passively forgotten.

However, compared to the nondepressed group, the depressed group had higher recall rates for negative items in both "Think" and "No-Think" conditions. This result suggests that depressed individuals not only excessively retrieved negative targets in response to the "Think" instruction, but also were less successful in memory suppression of negative targets when the "No-Think" instruction was given. Accordingly, ERP components of frontal N2 (reflecting voluntary memory inhibition) and parietal LPC (reflecting conscious recollection) showed deflection for negative (but not neutral) items in depressed compared with nondepressed, participants.

The N2 result suggested that depressed participants can hardly suppress the memory retrieval of negative materials. Previous studies manifested that depressed individuals were more difficult to exert cognitive control over unwanted thoughts, thus their recall rates of No-Think items were higher than nondepressed participants^7^,^8^,^10^,^24^. Going beyond the prior work, the current study statistically compared the memory suppression for negative and neutral materials, thus explicitly revealed that the deficit of memory inhibition was specifically pronounced for negative but not neutral stimuli in depression. More importantly, the N2 pattern in this study provided neural evidence for the mechanism underlying the memory inhibitory deficit in depressed population. In general, researchers proposed two potential mechanisms: 1) the deficit was due to that the efforts to suppress memory were less efficient in depressed than in nondepressed individuals^7^,^8^, which was supported by studies showing compensatory overactivation of LPFC and ACC during inhibition tasks in depressed patients^57^; 2) the deficit was caused by motivational absence and lack of effort in depression^8^,^25^; this mechanism was supported by the finding that lower cerebral blood flow was observed in frontal cortex and cingulate gyrus during depressive states^58^. Therefore, if the first proposal is true, the ERP component associated with cognitive control (i.e., the N2) should have larger amplitudes for negative "No-Think" items (low efficiency needs more compensatory resources). In contrary, if the second proposal is true, the N2 should have smaller amplitudes for negative "No-Think" items (low motivation results in less resources being allocated). As the first ERP-based T/NT study in depression, our result supports the second proposal, i.e., the reduced N2 for negative "No-Think" items indicated that depressed individuals have low motivation and make little effort to suppress negative items. However, this inference is not straightforward because the current task could not explicitly measure the motivation level and the cognitive control ability of the participants. To identify the two proposals, a further experiment with a more sophisticated design is suggested.

Another novel finding of this study was that compared with nondepressed group, the LPC amplitude in depressed group was larger for negative (but not neutral) items in Think condition. The LPC is an ERP marker of conscious recollection and is sensitive to retrieval attempts^36^,^59^; a larger amplitude of LPC may reflect an augment of recollection-related activity in the parietal-hippocampal network^17^,^23^. Our LPC result indicated that negative memories are more likely to be revisited by depressed participants (compared with nondepressed ones) due to their mood-congruent and intrusive nature. Preferential recall of negative compared with neutral and positive material is one of the most robust findings in the depression literature^60^. The current LPC finding is consistent with previous memory studies, which observed a larger LPC and a higher recall rate for negative materials in depressed, compared with nondepressed, individuals^61^,^62^. As mentioned in the introduction, none of the behavioral T/NT studies have found abnormal memory recall of "Think" items in depressed participants (see Hertel & Gerstle^8^; Hertel & Mahan^24^). One contribution of the present study is to provide a sensitive ERP index of "Think" performance for the titration of excessive negative memory retrieval in depressed individuals.

In addition, our behavioral result of the nondepressed group seems to support the data reported by Chen *et al*.^23^. In their study, Chen *et al*.^23^ examined whether cognitive control of memories differs between negative and neutral materials, and found that participants had greater difficulty in suppressing negative memories than neutral ones, i.e., the recall rate was higher for negative than neutral items in the "No-Think" condition. In contrary, Depue *et al*.^22^ found that both the facilitatory (Think condition) and inhibitory influences (No-Think condition) of cognitive control were larger for negative than neutral items, i.e., the recall rate was higher for negative "Think" items and lower for negative "No-Think" items, compared to neural "Think" and "No-Think" items respectively. These two previous studies proposed two possible mechanisms underlying the memory suppression for negative materials in healthy individuals. The first is that negative items or events can be better elaborated into memory and thus more difficult to be suppressed because negative materials are more salient and intrusive than neutral ones^63^,^64^. The second proposal is that greater cognitive control can be exerted over negative materials compared to neutral ones^65^. Our finding is consistent with the first proposal, although the data did not achieve the significant level (maybe because the nondepressed individuals were with a relatively high level of anxiety in this study).

Three limitations should be pointed out for an appropriate interpretation of the current result. First, this study, as well as previous T/NT studies that employed face-picture as cue-target combinations^19^,^22^,^23^, did not include a memory recall test using independent cues (the latter was suggested by Anderson *et al*.^17^ and Anderson & Green^16^). Therefore it is unclear whether the suppression-induced forgetting was really caused by an executive control process that inhibits/weakens the neural representation of the "No-Think" target itself, or alternatively, was due to the degraded association between the cue and the target (Anderson & Green^16^). Further experiments are needed to isolate the contribution of other mechanisms underlying the impaired memory suppression in depression.

Second, this study did not include a condition with positive material. When we examine the mechanisms behind suppression-induced forgetting for negative material, a positive condition would help tease apart whether the ERPs are reflective of cognitive control for emotional memories in general (anything with emotional salience) or specifically negative memories. Thus, whether depressed individuals would demonstrate enhanced memory for positive material in the T/NT task due to shared saliency with negative material has not been ruled out due to the lack of a positive condition.

Third, this study considered the undergraduate students scored >13 in BDI-II as depressed subjects. Thus the 25 participants in depressed group were very likely below the clinical levels of major depressive disorder. Furthermore, because anxiety and depressive symptoms are highly comorbid, this study only selected participants with high-trait anxiety in depressed and nondepressed groups.

In conclusion, by comparing the performances and brain activities between depressed and nondepressed participants, we identified two electrophysiological effects associated with memory facilitation and suppression. Both the dysfunctions of memory control, i.e., excessive attempts to retrieve negative items (reflected by enhanced LPC) and deficient efforts to avoid retrieval of negative events (reflected by reduced N2), contribute to the difficulties in forgetting negative material in depression. Our findings extended previous T/NT studies that used only behavioral measures, and provided more empirical evidences that may help to understand the mechanism underlying the onset and maintenance of depressive disorders.

## Additional Information

**How to cite this article**: Zhang, D. *et al*. Neural correlates underlying impaired memory facilitation and suppression of negative material in depression. *Sci. Rep*. **6**, 37556; doi: 10.1038/srep37556 (2016).

**Publisher’s note:** Springer Nature remains neutral with regard to jurisdictional claims in published maps and institutional affiliations.

## Figures and Tables

**Table 1:** Demographic data of depressed and nondepressed participants.

Characteristics	depressed (n = 25)	nondepressed (n = 25)	Statistics
Mean age, y	19.2 (17–21)	18.7 (17–20)	*t*(48) = −1.77, *p* = 0.083
Sex, male/female	12/13	13/12	
Handedness, right/left	25/0	25/0	
BDI-II	20.8 (15–28)	4.96 (0–12)	*t*(48) = −14.6, *p* < 0.001
STAI-T	55.6 (50–64)	57.0 (50–67)	*t*(48) = −1.14, *p* = 0.259

Descriptive data are presented as mean (range).

BDI-II, Beck Depression Inventory (Second Edition).

STAI-T, the Trait form of Spielberger's State-Trait Anxiety Inventory.
